# Genomic-, phenotypic-, and toxicity-based safety assessment and probiotic potency of *Bacillus coagulans* IDCC 1201 isolated from green malt

**DOI:** 10.1093/jimb/kuab026

**Published:** 2021-04-27

**Authors:** Won Yeong Bang, O-Hyun Ban, Bo Som Lee, Sangki Oh, Chanmi Park, Mi-Kyung Park, Sung Keun Jung, Jungwoo Yang, Young Hoon Jung

**Affiliations:** School of Food Science and Biotechnology, Kyungpook National University, Daegu 41566, Republic of Korea; Ildong Bioscience, 17 Poseunggongdan-ro, Pyeongtaek-si, Gyeonggi-do 17957, Republic of Korea; Ildong Bioscience, 17 Poseunggongdan-ro, Pyeongtaek-si, Gyeonggi-do 17957, Republic of Korea; School of Food Science and Biotechnology, Kyungpook National University, Daegu 41566, Republic of Korea; Ildong Bioscience, 17 Poseunggongdan-ro, Pyeongtaek-si, Gyeonggi-do 17957, Republic of Korea; Ildong Bioscience, 17 Poseunggongdan-ro, Pyeongtaek-si, Gyeonggi-do 17957, Republic of Korea; School of Food Science and Biotechnology, Kyungpook National University, Daegu 41566, Republic of Korea; School of Food Science and Biotechnology, Kyungpook National University, Daegu 41566, Republic of Korea; Ildong Bioscience, 17 Poseunggongdan-ro, Pyeongtaek-si, Gyeonggi-do 17957, Republic of Korea; School of Food Science and Biotechnology, Kyungpook National University, Daegu 41566, Republic of Korea; Institute of Fermentation Biotechnology, Kyungpook National University, Daegu 41566, Republic of Korea

**Keywords:** Probiotics, *Bacillus coagulans*, Safety evaluation, Genomic safety, Toxicity, Property

## Abstract

Probiotics are beneficial microorganisms, and the evaluation of their safety for human use in the food industry has become critical. This study examines the safety of *Bacillus coagulans* IDCC 1201 isolated from green malt by analyzing its genomic and phenotypic characteristics and determining its toxicity. The presence of antibiotic resistance and toxigenic genes and gene transferability were investigated using whole-genome analysis. The strain's hemolytic and enzyme activities, minimum inhibitory concentrations of antibiotics, and biogenic amine and D-lactate production were also examined. Furthermore, the principal properties of *B. coagulans* IDCC 1201 as probiotics, such as resistance to abiotic stress and intestinal adhesion, were studied. The whole-genome analysis demonstrated that *B. coagulans* IDCC 1201 had no antibiotic resistance or toxigenic genes; the strain was susceptible to the nine antibiotics proposed by the European Food Safety Authority. Moreover, this strain lacked hemolytic and β-glucuronidase activities. Additionally, it was confirmed that *B. coagulans* IDCC 1201 produced undesirable metabolites, including biogenic amines or D-lactate, at a safe level. Finally, the strain exhibited functional potential as a probiotic in terms of abiotic tolerance, such as bile tolerance and intestinal adhesion in *in vitro* experiments. In conclusion, *B. coagulans* IDCC 1201 can be considered as a safe probiotic with regard to human health.

## Introduction

*Bacillus coagulans* is a Gram-positive, nonpathogenic, endospore-forming, facultative anaerobic, and rod-shaped bacterial species (Forrester & Wicken, 1966). Initially identified as *Lactobacillus sporogenes*, it was first isolated from spoiled milk in 1933 (André et al., 2017). Unlike other *Bacillus* species, *B*. *coagulans* does not produce endotoxins and is generally considered harmless (Lee & Salminen, 1995). As a result, *B. coagulans* strains, for example, LactoSpore and GBI-30, have been listed as Generally Recognized as Safe (GRAS) by the U.S. Food and Drug Administration (FDA) since 2012.

In addition, the U.S. FDA and the European Food Safety Authority (EFSA) have suggested the use of *B. coagulans* as a probiotic food supplement (Konuray & Erginkaya, 2018). Today, *B. coagulans* is considered a promising probiotic due to its characteristics, such as tolerance for bile salt and acid, that enable its high survivability in the gastrointestinal tract (Zhou et al., 2020). Furthermore, its probiotic properties have been demonstrated to alleviate irritable bowel syndrome (Majeed et al., 2015) and bacterial vaginosis (Sudha & Bhonagiri, 2012).

Meanwhile, the safety of probiotics has become the most important criterion beyond its functional efficacy for human health in the food industry (Salvetti et al., 2016). Several studies have evaluated the safety of *B. coagulans* strains at the phenotypic assay level or genomic level (Salvetti et al., 2016; Li et al., 2018; Kapse et al., 2019; Saroj & Gupta, 2020). However, the attributes and health-related properties of probiotics are strain-specific (Salvetti et al., 2016; Kapse et al., 2019). As a result, a recent study has suggested a workflow to assess the genomic as well as phenotypic safety of a probiotic strain to increase the reliability of safety (Salvetti et al., 2016).

In the present study, the presence of antibiotic resistance and virulence genes and gene transferability of *B. coagulans* IDCC 1201 were investigated using whole-genome analysis. Second, the phenotypes of *B. coagulans* IDCC 1201, including hemolytic and enzymatic activities, minimum inhibitory concentration (MIC) tests, and biogenic amine and l-/d-lactate production, were characterized. Then, the cytotoxicity of this strain was investigated both *in vitro* and *in vivo*. Finally, the various probiotic properties, including intestinal adhesion and tolerance against abiotic stresses, were explored. Therefore, we believe that this study contributes valuable criteria to the assessment of other *B. coagulans* strains.

## Materials and Methods

### Culture Conditions of *B. coagulans*

*B. coagulans* IDCC 1201 (CP035305, GenBank), isolated from green malt, was cultured in MRS medium (BD Difco, Franklin Lakes, NJ, USA) without agitation at 37°C for 24 hr. MRS medium consists of 1% peptone, 1% beef extract, 0.4% yeast extract, 2% dextrose, 0.1% polysorbate 80, 0.2% ammonium citrate, 0.5% sodium acetate, 0.02% magnesium sulfate, 0.005% manganese sulfate, and 0.2% dipotassium phosphate. Cell growth was measured at 600 nm using a microplate spectrophotometer (EPOCH2, BioTek, Winooski, VT, USA). For spore formation of *B. coagulans* IDCC 1201, the strain was grown without sugar sources.

### Genomic Analysis of Safety-Related Genes in *B. coagulans*

#### Whole-genome sequencing

Whole-genome sequencing of *B. coagulans* was performed using the PacBio RSII instrument on the Illumina platform (Macrogen, Seoul, Korea). A nucleotide sequence was generated by DNA polymerase with a circular single-molecule real-time sequencing system. The polymerase reads were trimmed to include only the high-quality regions, the sequences from the adapters, and the sequences from multiple passes around a circular template. Each polymerase read was partitioned to form one or more subreads to contain the sequences from a single pass of a polymerase on a single strand of an insert within a SMRTbell™ template and no adapter sequences.

The sequencing library was prepared by the random fragmentation of the DNA or the cDNA sample, followed by 5′ and 3′ adapter ligation. This library was loaded into a flow cell where the fragments were captured on a lawn of surface-bound oligos complementary to the library adapters. As all four reversible, terminator-bound dNTPs were present during each sequencing cycle, the natural competition minimizes incorporation bias, significantly reducing the raw error rates compared to other technologies. Then, the sequencing data were converted into raw data for analysis. Contigs were constructed by *de novo* assembly using the RS HGAP Assembly software (v. 3.0), which initially preassembled the seed reads, generated a consensus sequence of the mapped reads, and corrected and filtered the reads. Next, the HiSeq reads were applied in the error–correction of the constructed contigs by Pilon (v. 1.21). Then, a consensus sequence with higher quality was obtained through self-mapping. The whole-genome sequences data have been deposited into NCBI with accession no. CP035305.1.

#### Phylogenetic analysis

Protein-encoding genes were predicted using Prodigal v.2.6.3 (Hyatt et al., 2010). Ribosomal RNA, transfer RNA, and various other genetic features were predicted using Rfam v12.0 (Griffiths-Jones, 2007). Orthologs were identified using the OrthoMCL program with an inflation value of 3.0 (Li et al., 2003). Duplicated genes of the core gene sets were excluded for the construction of the phylogenomic tree. The amino acid sequences of each ortholog were aligned with MUSCLE v3.8.31 (Edgar, 2004); aligned positions with more than 50% gaps were removed using Gblocks v0.91 (Castresana, 2000). The final gene alignments were concatenated using FASconCAT (Kück & Meusemann, 2010). The best-fitting substitution models were determined using Model Test-NG v0.1.6. Using the Akaike information criteria and the Bayesian information criteria (Darriba et al., 2020). Protein sequence alignments were converted into the corresponding codon alignments using the PAL2NAL program (Suyama et al., 2006). The phylogenomic tree of the *B. coagulans* group based on coding sequences was constructed with RAxML using the model GTRGAMMAIX to select for strains of *B. coagulans* Group II and GTRGAMMAX to select for closely related strains to IDCC 1201, selected by Model Test-NG v0.1.6.

#### Evaluating genomic safety using virulence factors and antibiotic resistance genes

Putative virulence factors and antibiotic resistance genes were explored using a local protein–protein basic local search tool (BLASTn) against the virulence factor database (VFDB; v. 2020.02.13; http://www.mgc.ac.cn/VFs/main.htm) (Chen et al., 2005) and the antibiotic resistance genes database (ARDB; http://ardb.cbcb.umd.edu/) (Liu & Pop, 2009), respectively. The thresholds showing more than 70% identity, more than 70% coverage, and an *E*-value of less than 1E^−5^ were considered positive (https://www.nature.com/articles/srep41259). Furthermore, the presence of acquired antimicrobial resistance genes was confirmed using the ResFinder program and database (https://cge.cbs.dtu.dk/services/ResFinder/) (Zankari et al., 2012).

#### Predicting genome stability using mobile elements and genomic islands

Putative prophage regions were identified using the PHAge Search Tool Enhanced Release (PHASTER) web-based program (http://phaster.ca/) (Arndt et al., 2019). Transposases and conjugal transfer proteins were annotated using BLASTP against the transposases and conjugal transfer proteins retrieved from GenBank. The genomic islands were predicted using the genomic island prediction software (GIPSy program; https://www.bioinformatics.org/groups/?group_id=1180) (Soares et al., 2016).

#### Predicting biogenic amine production and D-lactate production

It was reported that histidine decarboxylase, tyrosine decarboxylase, lysine decarboxylase, ornithine decarboxylase, phenylalanine decarboxylase, and the enzymes involved in the agmatine deiminase pathway, such as *N*-carbamoylputrescine amidase and agmatinase, were critical for the biosynthesis of biogenic amines (Ruiz-Capillas & Jiménez-Colmenero, 2010; Gardini et al., 2016). Further, the enzymes involved in the biosynthesis of D-lactate from l-lactate, methylglyoxal, and pyruvate have been identified to be lactate racemase, hydroxyacylglutathione hydrolase, D-lactate dehydratase, D-lactate dehydrogenase, and 2-hydroxyglutarate-pyruvate transhydrogenase (Garvie, 1980; Creighton et al., 1988; Taguchi & Ohta, 1991; Becker-Kettern et al., 2016). We downloaded the sequences of the genes encoding these enzymes from the UniProtKB database and built profile HMMs representing the conserved amino acid sequence patterns in these enzymes. The candidate genes in the *B. coagulans* IDCC 1201 genome involved in biogenic amine biosynthesis were searched using the *hmmsearch* tool in the HMMER package (Finn et al., 2011) with the constructed profile HMMs. The candidate genes were confirmed using BLASTP in NCBI's BLAST+ and the *hmmscan* tool in the HMMER package against the corresponding genes in the SWISS-PROT and PFAM databases.

### Safety Assessment of *B. coagulans*

#### Hemolysis test

The ability to rupture red blood cells is the primary characteristic of pathogenic bacteria. *B. coagulans* IDCC 1201 grown in MRS broth overnight was streaked onto sheep blood agar plates (BD Difco), and incubated at 37°C until clear zones around the colony observed. *Staphylococcus aureus* subsp. *aureus* (ATCC 25923) and *Lactobacillus reuteri* were used as the positive control for β-hemolysis and negative control for γ-hemolysis, respectively. *S. aureus* was incubated in brain heart infusion medium at 37°C, while *L. reuteri* was grown in MRS broth at 37°C and 100 rpm.

#### Enzymatic activity test

Nineteen types of enzymatic activities of *B. coagulans* IDCC 1201 were investigated using the API ZYM Kit (bioMérieux, Marcy l'Etoile, France) according to the manufacturer's instruction. In short, 1.8 × 10^9^ colony forming units (CFUs)/ml of *B. coagulans* IDCC 1201 were loaded into an API ZYM strip already activated in MRS medium at 37°C for 4 hr. After a 5-min reaction with ZYM A and ZYM B, the color changes indicating enzymatic activities were evaluated according to the color reaction chart.

#### Antibiotic susceptibility examination

The antibiotic susceptibility of *B. coagulans* IDCC 1201 was determined based on MIC values. In short, approximately 1–2 × 10^8^ CFU/ml of *B. coagulans* IDCC 1201 was spread onto each MRS agar plate. Then antibiotic (*E*-test) strips containing ampicillin, chloramphenicol, clindamycin, erythromycin, gentamicin, kanamycin, streptomycin, tetracycline, and vancomycin (Liofilchem, Waltham, MA, USA) were placed on the agar plates.

Cell growth inhibition was also investigated to confirm the results of the antibiotic susceptibility test. *B. coagulans* IDCC 1201 at 10^6^ CFU/ml and 1:1 (v/v) to each antibiotic solution at various concentrations were transferred to a 96-well plate and incubated at 37°C for 20 hr. Then, the optical density of each incubation was observed for 20 hr using a microplate reader (BioTek, Winooski, VT, USA). The cutoff values of the MICs were determined according to EFSA's technical guidelines of the EFSA on antibiotic susceptibility (EFSA, 2018).

#### Biogenic amine production test

The production of biogenic amines, such as tyramine, histamine, putrescine, cadaverine, and 2-phenylethylamine, by *B. coagulans* IDCC 1201 was investigated. After culturing *B. coagulans* for 24 hr at 37°C, 1.5 ml of the culture broth was centrifuged at 12 000 rpm for 5 min at 4°C to obtain a cell-free supernatant. After mixing the supernatant with 0.75 ml of 0.1 N HCl, 1 ml of the filtered mixture was incubated with 200 µl of saturated NaHCO_3_, 20 µl of 2 M NaOH, and 0.5 ml of dansyl chloride (Sigma-Aldrich, St. Louis, MO, USA). After the derivatization reaction, 200 µl of proline was added. The reaction was then vortexed for 1 min, incubated in the dark at room temperature for 15 min, and stopped by adding acetonitrile (HPLC-grade; Sigma-Aldrich) of up to 5 ml. Next, each of the derivatized amines was analyzed by high-performance liquid chromatography (HPLC; Jasco LC-NETOII/ADC, Jasco, Macclesfield, UK) equipped with a wavelength detector (Jasco) on a 4.6 mm × 250 mm, 5-µm Athena C18 column with a pore size of 120 Å using aqueous acetonitrile (67 vol%) as the mobile phase with a constant flow rate of 0.8 ml/min. The amines were quantified at 254 nm.

#### Lactate measurement

The optical purity of the resultant lactic acid was estimated using an l-/d-lactate enzymatic test kit following the manufacturer's instructions (Megazyme, Bray, Ireland). In brief, cell-free supernatants were obtained from overnight cultures of *B. coagulans* IDCC 1201 after centrifugation at 7 000 rpm and 4°C for 30 min and assayed using l-/d-lactate dehydrogenase and glutamate-pyruvate transaminase. Then, the absorbance of diluted supernatant was measured at 340 nm, and the l-/d-lactate concentrations were calculated according to the manufacturer's protocol.

### Toxicity Measurements of *B. coagulans*

#### Cytotoxicity

A549 (adenocarcinomic human alveolar basal epithelial) cells at 1.2 × 10^5^ cells/ml), HUVECs (human umbilical vein endothelial cells at 1 × 10^4^ cells/ml, and HaCaT (spontaneously transformed aneuploid immortalized keratinocyte derived from adult human skin) cells at 2 × 10^5^ cells/ml were seeded on 96-well plates. When the cells reached 70%–80% confluency, the medium was replaced with 100 µl of medium containing *B. coagulans* to incubate for 24 hr. Twenty microliter of the MTS/PMS mixture (Promega, Madison, WI, USA) was added to the A549 cells and HUVECs to measure cell viability using a microplate reader at the absorbance of 490 nm (Bio-Rad, Hercules, CA, USA). On the other hand, 10 µl of the 5 mg/ml 3-(4,5-dimethyl-2-thiazolyl)-2,5-diphenyl-2*H*-tetrazolium bromide solution (Sigma-Aldrich) was added for the HaCaT cells in each well. After 4 hr, the supernatant in each well was discarded, and the formed crystals were dissolved in 100 µl of dimethyl sulfoxide (Sigma-Aldrich). After shaking for 15 min, cell viability was measured using a microplate reader at the absorbance of 590 nm.

#### Acute toxicity in rats

Acute oral toxicity (AOT) test was performed by the Korea Testing and Research Institute (KRT; TGK-2019-000170; Hwasun-gun, Jeollanam-do, Korea). The AOT test was performed according to the test guidelines of the Animal Protection Act (no. 14651) and Laboratory Animal Act (no. 15278) by the Korean government. Female rats were randomly divided into four groups of three; two groups were 9 weeks old, and the other two were 10 weeks old. The rats in the treatment group were orally administered with 10 ml of *B. coagulans* at 8.5 × 10^10^ CFU/g at 300 mg/kg body weight (BW) or 2 000 mg/kg BW once daily for 14 days. All rats were observed for clinical signs of morbidity and mortality and measured for BW during the treatment period and after dosing ended. Afterward, all the rats were sacrificed to examine organs under isoflurane anesthesia. All the experiments were conducted with the aim to minimize animal suffering and distress and the number of animals used while obtaining reliable scientific data.

### Identification of Probiotic Properties of *B. coagulans*

#### Carbohydrate fermentation patterns

The carbohydrate fermentation patterns of *B. coagulans* IDCC 1201 were investigated using an API 50 CHL/CHB kit (bioMérieux) and 49 selected carbohydrate sources according to the manufacturer's guidelines. In brief, the bacteria grown in MRS medium overnight were suspended in 10 ml of API 50 CHL medium and applied to cupules containing different carbohydrates on an API 50 CH test strip. The fermentation patterns were monitored for up to 48 hr at 37°C.

#### Heat tolerance

Overnight-grown *B. coagulans* IDCC 1201 cultures at 2.6 × 10^9^ CFU/ml were heated in a water bath at 70°C for 20, 40, or 80 s. Afterward, 100 µl of each sample was spread onto an MRS agar plate to measure the viable cell count.

#### Acid tolerance

Overnight-grown *B. coagulans* IDCC 1201 cultures were centrifuged at 8 000 × *g* for 5 min, and the cell pellets were washed twice with sterile 1 × PBS buffer (pH 7.3). Then, 100 µl of 10 × concentrated cells were inoculated into 10 ml of PBS whose pH had been adjusted to 1, 2, 3, and 4 using 1 M hydrogen chloride. The cells at 1.0 × 10^8^ CFU/ml were incubated at 37°C for 3 hr; at each hour, 100 µl were collected from each reaction and spread onto an MRS agar plate to measure the viable cell count.

#### Bile tolerance

*B. coagulans* IDCC 1201 was cultured overnight and centrifuged at 8000 × *g* for 5 min. A 10× concentrated cell pellet was then incubated in a solution with the final concentration of 0.3% (wt/vol) bile salt (OX gall/OX bile). The diluted solution at the cell concentrations of 2.6 × 10^9^ CFU/ml was incubated at 37°C for 6 hr; 100 µl were collected from each reaction and spread on an MRS agar plate to measure the viable cell count.

#### Hydrophobicity and autoaggregation

*B. coagulans* IDCC 1201 grown overnight was centrifuged at 8000 × *g* for 15 min at 4°C, and the cell pellets were washed twice with and resuspended in PBS buffer to an OD of 0.5 at 600 nm. Hydrophobicity was measured by mixing 3 ml of the cell suspension with 0.6 ml of *n*-hexadecane, vortexing thoroughly for 2 min, incubating at 37°C for 2 hr without mixing, and measuring the aqueous phase, or the lower layer of the two separated phases, at the absorbance of 600 nm.

Autoaggregation was measured by incubating 12 ml of the cell suspension (OD 0.5) at 37°C for 5 hr and sampling at hours 0, 1, 3, and 5. After incubation at 37°C for 5 hr without mixing, the absorbance of the upper suspension was measured at 600 nm. The percentages of hydrophobicity were expressed as the following:

$$\begin{equation*}
{\rm{Hydrophobicity}}\,\left( \% \right)\!:\!\left( {A_0} - {A_t}\right)/{A_0} \times 100
\end{equation*}$$

with *A_t_* representing the absorbance at time *t* = 0, 2 hr, and *A*_0_ indicating the absorbance at *t* = 0 hr.

The percentages of autoaggregation were calculated as the following:

$$\begin{equation*}
{\rm{Autoaggregation}}\,\left( \% \right)\!:\!(1 - {A_t}/{A_0}) \times 100
\end{equation*}$$

with *A_t_* representing the absorbance at time *t* = 0, 1, 3, 5 hr, and *A*_0_ indicating the absorbance at *t* = 0 hr.

## Results and Discussion

### Genomic Analysis of the Safety-Related Genes

**Fig. 1:**
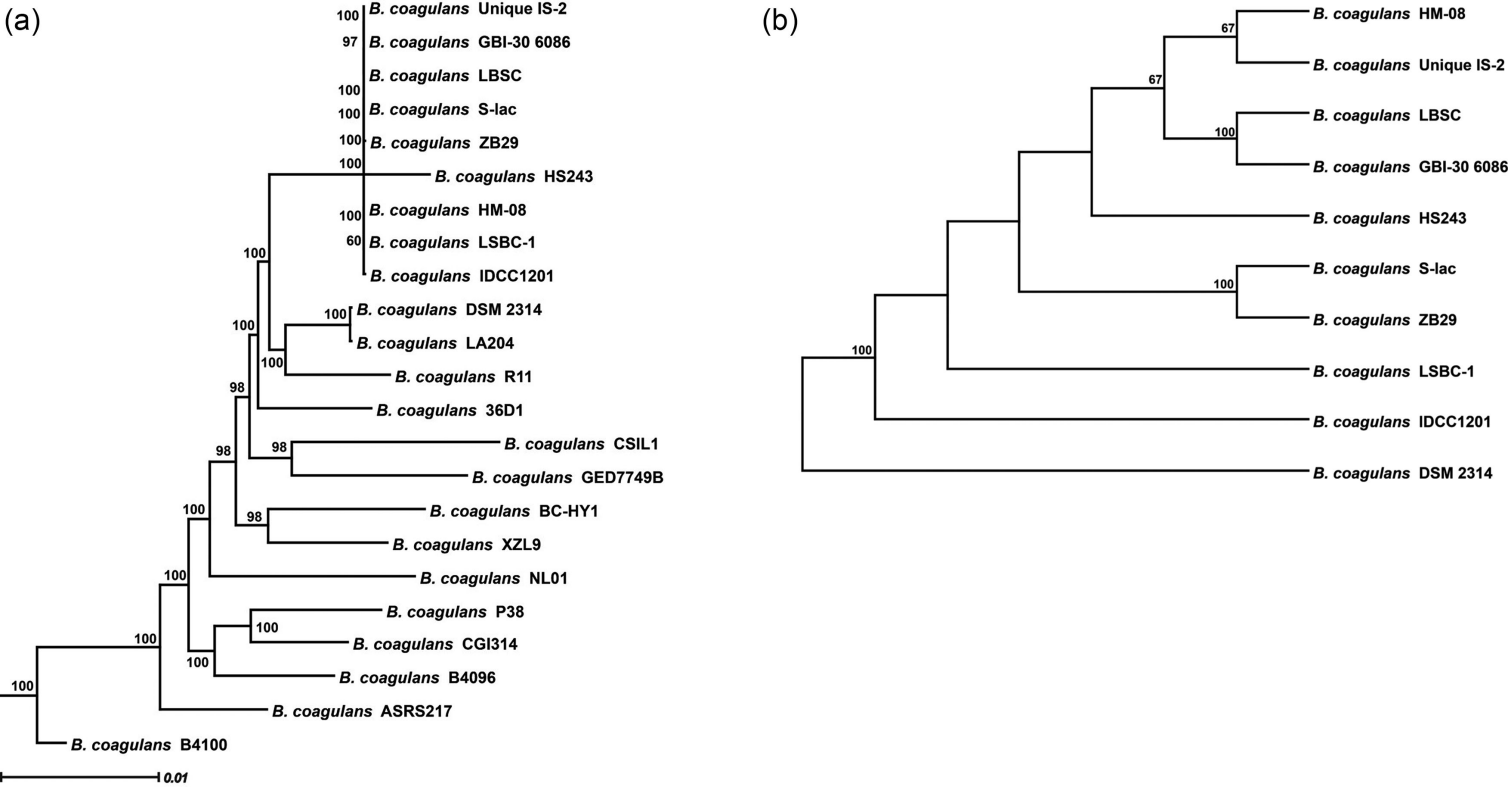
The phylogenomic tree of the strains belongs to subgroup II of *B. coagulans* (a) and closely related strains to *B. coagulans* IDCC 1201 (b). The tree was constructed using the coding sequence alignments of 2 083 core genes with the maximum likelihood approach. Numbers above the branches demonstrated maximum likelihood bootstrap supports from 500 nonparametric replicates (shown only if they were >0.5). The scale bar represents the number of substitutions per site.

Genome analysis confirmed the strain used in this study to be *B. coagulans* containing 3.6 million base pairs of the genome with a 46.3% GC content (Supplementary Table S1 and Fig. S1). Thirty-six sequenced genomes were retrieved from the NCBI genome database (Supplementary Table S2) for the comparative genome study of *B. coagulans* IDCC 1201. The genome sequences of *B. acidiproducens* DSM 23148 and *B. shackletonii* LMG 18435 were used as outgroups in the phylogenomic analysis. As a result, the tree defined two discrete subgroups of *B. coagulans* (Supplementary Fig. S2). Then, the tree was reconstructed based on the coding sequence alignments of 2083 core genes using the maximum likelihood approach. It was revealed that the tree was rooted by *B. coagulans* ATCC 7050 and 2–6 outgroups (Fig. 1a). Finally, the tree was reconstructed using the coding sequence alignments of 2 781 core genes using the maximum likelihood approach to illustrate the evolutionary root of the outgroup containing *B. coagulans* IDCC 1201. *B. coagulans* DSM 2314 was found to be the evolutionary root of subgroup II (Fig. 1b). Interestingly, *B. coagulans* DSM 2314, IDCC 1201, LSBC-1, and IS-2 were isolated from Japan, Korea, China, and India, respectively. Thus, the *B. coagulans* strains in this subgroup were likely to have mainly evolve from Eastern to Western Asia, regardless of their source.

Various methodologies based on genomic information, such as identifying the virulence factor (Salvetti et al., 2016), evaluating the antibiotic resistance genes (Bernardeau et al., 2008; Kiwaki & Sato, 2009), and predicting genetic stability (Bhardwaj et al., 2010), were performed to evaluate the safety of probiotic strains systematically. No putative virulence factors were found in *B. coagulans* IDCC 1201 through BLASTn analysis against the VFDB dataset (Table 1). The genomic stability of *B. coagulans* IDCC 1201 was confirmed by analyzing its mobile elements and genomic islands. The entire genome contained 95 genes encoding transposases, but no transposase was found in the 10-kb regions surrounding the virulence genes. Also, no genes involved in conjugal transfer were found in the genome of *B. coagulans* IDCC 1201, indicating a low risk of gene transfer. While there were three prophage regions in the chromosome (Supplementary Table S3), they were not associated with the virulence or antibiotic resistance genes. Additionally, 16 genomic islands were predicted in the whole-genome using *B. coagulans* DSM 2314’s genome sequence as the reference (Supplementary Table S4). Among the genomic islands, GI_12 contained a gene encoding the aforementioned virulence factor (1201_1_01351) commonly found in the genomes of *Bacillus* spp. (Saroj & Gupta, 2020). In addition, the amino acid sequence encoded by that gene was identical to that of a corresponding protein in the genome of *B. coagulans* S-lac, which is a commercially available probiotic strain. These results suggest *B. coagulans* IDCC 1201 is considered safe and may not possess transferrable genes, including antibiotic-related genes.

**Table 1.** Genes Related to (A) Virulence Factors, (B, C) Antibiotic Resistance, and (D) Biogenic Amines

Gene ID	Contig name	Start	End	Strand	Subject ID	Identities	Query coverage	VFID	VF name				
–	–	–	–	–	–	–	–	–	–				
B													
Contig_name^a^	Gene_ID	Start	End	Query coverage	Identity	Cut_off Identity value	Subject_ID	Subject coverage	Gene_name	Related antibiotics			
–	–	–	–	–	–	–	–	–	–	–			
C													
Gene ID^b^	Contig_name	Start	End	Strand	Cut_Off	Pass bitscore	Best_Hit bitscore	Best_Hit ARO	Best Identities	Model_type	Drug Class	Resistance mechanism	AMR gene family
–	–	–	–	–	–	–	–	–	–	–	–	–	–
D													
Locus tag	Gene	Identity	Homology to	PFAM domain									
IDCC 1201_1_01385	hdcA	86.8	histidine decarboxylase of *Lactobacillus* sp. A30 (AAB59151)	Histidine decarboxylase (PF02329)									

^a^Antibiotic resistance genes were searched by ARDB using cutoff values of gene families.

^b^Antibiotic resistance genes were searched by CARD with strict significance.

### Hemolytic Properties and Enzymatic Activities

In general, hemolytic activity is caused by pathogenic bacteria, including several Gram-positive bacteria (Nakajima et al., 2003). Hence, a lack of hemolytic activity is an important criterion of safety for the selection of probiotics. In the present study, *B. coagulans* IDCC 1201 did not exhibit any hemolytic activity, that is, γ-hemolysis, when cultured on sheep blood agar (Table 2). In contrast, *S. aureus* subsp. *aureus*, which was used as the positive control, induced β-hemolysis. On the other hand, *L. reuteri*, which was used as the negative control, displayed no hemolytic activity.

**Table 2.** Hemolytic Activities of *B. coagulans* IDCC 1201

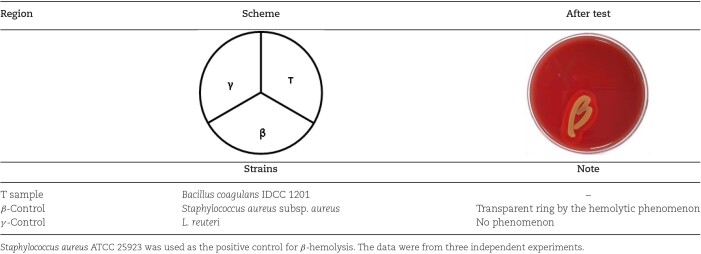

Studying the production of microbial enzymes, such as β-glucuronidase that triggers mutations and increases carcinogenicity (Kim & Jin, 2011; Zhu et al., 2013; Jung et al., 2019) and β-glucosidase that generates carcinogenic aglycones and causes colorectal cancer (Allsopp & Rowland, 2009; Ji et al., 2015), is also a critical parameter for assessing the safety of a bacterial strain. Here, the production of β-glucuronidase and β-glucosidase by *B. coagulans* IDCC 1201 was not detected (Supplementary Table S5). No other specific threats were observed during the profiling of its enzymatic activities.

### Determination of the Antibiotic Resistance and Biogenic Amine and Lactate Production of *B. coagulans* IDCC 1201

Although antibiotic resistance genes were not identified in the genomes of *B. coagulans* IDCC 1201 (Table 1), the safety of *B. coagulans* needed to be further confirmed by the *in vitro* analysis of its MICs of various antibiotics. *B. coagulans* IDCC 1201’s antibiotic susceptibility was analyzed using E-strips containing nine antibiotics, including inhibitors of cell-wall synthesis like ampicillin and vancomycin and inhibitors of protein synthesis like chloramphenicol, clindamycin, erythromycin, gentamicin, kanamycin, streptomycin, and tetracycline (Table 3). All the antibiotics tested exhibited a MIC range of 0.023–1.0 mg/l by *E*-tests and of 0.125–8 mg/l by cell growth tests, indicating that *B. coagulans* is susceptible to these antibiotics (Majeed et al., 2016). These results were highly comparable to those of *B. coagulans* MTCC 5856, one of the GRAS strains (Majeed et al., 2015).

**Table 3.** The Minimum Inhibitory Concentrations of *Bacillus coagulans* IDCC 1201 Against Various Antibiotics

	AMP	CHL	CLI	ERY	GEN	KAN	STR	TET	VAN
	n.r.	4	4	8	8	4	4	8	8
*E*-test^a^									
Concentration (mg/l)	0.019	1.0	0.023	0.094	0.064	0.5	0.75	0.032	0.094
Susceptibility	n.r.	S	S	S	S	S	S	S	S
Growth test									
Concentration (mg/l)	0.5	4–8	<0.125	<0.125	<1	4	4–8	<0.125	<1
Susceptibility	n.r.	S	S	S	S	S	S	S	S
*B. coagulans* MTCC 5856^b^	0.062	1.0	0.0078	0.125	0.062	1.0	1.0	0.062	0.25

Abbreviations: n.r.: not required, R: resistant, S: susceptible; AMP, ampicillin; CHL, chloramphenicol; CLI, clindamycin; ERY, erythromycin; GEN, gentamicin; KAN, kanamycin; STR, streptomycin; TET, tetracycline; VAN, vancomycin.

^a^EFSA (2018).

^b^Majeed et al. (2016).

Biogenic amines are generally produced by pathogenic bacteria and some lactic acid bacteria by converting amino acids into basic and nitrogenous compounds (Beneduce et al., 2010; Spano et al., 2010; Barbieri et al., 2019). These amines are involved in a wide range of natural physiological processes; however, large quantities of biogenic amines can lead to undesirable effects such as vomiting and diarrhea (Beneduce et al., 2010; Barbieri et al., 2019).

The production of biogenic amines by microorganisms is highly variable and strain-specific. *B. coagulans* IDCC 1201’s genome was searched for genes related to the biosynthesis of biogenic amines, such as tryptamine and putrescine and was found to harbors one gene (ESP47_12915) encoding an agmatinase, a catalyst for converting agmatine into putrescine and urea. The gene is highly similar, at 81.0%, to the reference sequence, that is, P70999 from *Bacillus subtilis* strain 168 (GenBank Acc. No. NP_391629). However, the genes encoding other biosynthesis-related enzymes, including the tyrosine decarboxylase, were not presented in this genome. These results suggest that the strain has the potential activity for the biosynthesis of the putrescine. However, unlike other lactobacillus species known for robust biogenic amine production, the five major biogenic amines, including cadaverine, histamine, tyramine, putrescine, and spermidine, were not produced by *B. coagulans* IDCC 1201 (Table 4). If the strain produces a large amount of biogenic amine during fermentation, it is challenging to prevent amine accumulation in the final fermented products. Thus, *B. coagulans* IDCC 1201 could be utilized as a safe food microorganism due to its biogenic amine production.

**Table 4.** Biogenic Amine and Lactic Acid Production by *B. coagulans*

	Cadaverine	Histamine	Putrescine	Spermine	Tyramine
	l-lactic acid	D-lactic acid	Ratio of isomers		
			l-form	D-form	
Concentration (mg/l)	ND	ND	ND	ND	ND
Concentration (mg/l)	4.73 ± 0.17	0.29 ± 0.00	94.22	5.78	

ND, not detected.

*B. coagulans*, classified as a *Lactobacillus* species, may also produce considerable amounts of lactic acid during fermentation (Ma et al., 2014). Both l- and D-lactate are present and metabolized in the human body; D-lactate, responsible for the lactate acidosis in the colon, usually is present in relatively small amounts at 5–20 µmol/l (Nielsen et al., 2011; Papagaroufalis et al., 2014; Vietta et al., 2017). The genome of *B. coagulans* IDCC 1201 was searched for genes related to the biosynthesis or bioconversion of D-lactate using HMMER and BLAST algorithms and was found to harbor a gene (ESP47_01620) encoding D-lactate dehydrogenase. The translated sequence contains a D-isomer specific 2-hydroxyacid dehydrogenase domain (PF02826) found in D-lactate dehydrogenases, suggesting that the strain has the potential to synthesize D-lactate. The lactate production by *B. coagulans* IDCC 1201 was observed to be 94.2% l-lactic acid and 5.8% D-lactic acid (Table 4). These data are consistent with previous research that *Bacillus* strains contained D-lactate dehydrogenase genes and produced small amounts of D-lactate; on the other hand, l-lactate constituted more than 99% of the total lactate production (Majeed et al., 2016). Thus, the *in vitro* tests confirm *B. coagulans* IDCC 1201’s safety in its antibiotic susceptibility and the lack of production of harmful metabolites.

### Cytotoxicity and *In Vivo* Single-Dose Acute Oral Toxicity Study

**Fig. 2:**
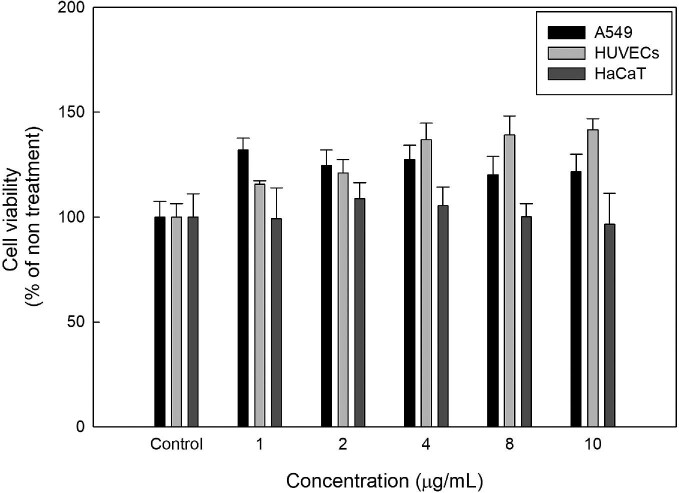
The measurement of *B. coagulans* IDCC 1201’s cytotoxicity on A549, HUVECs, and HaCaT cells. *B. coagulans* IDCC 1201 exhibited no detectable cell cytotoxicity in A549, HUVECs, and HaCaT cells.

Probiotic strains should not be deleterious; thus, it is necessary to evaluate their bacterial toxicity before use. First, the cytotoxicity induced by *B. coagulans* IDCC 1201 on different cells, that is, A549 cells, HUVECs, and HaCaT cells, was determined (Fig. 2). The percentage of viability varied slightly among the cell types. HaCaT cells had the lowest viability at approximately 90.5%. *B. coagulans* IDCC 1201 did not affect the viability of A549 cells and HUVECs in the tested range. Therefore, *B. coagulans* was not toxic to any of the three cell types.

The toxicity of *B. coagulans* IDCC 1201 was again tested in an *in vivo* single-dose AOT study. *B. coagulans*, at 8.5 × 10^10^ CFU/g, was fed to rats at a daily single oral dose of 0.3 or 2 g of per kg BW for 14 days. No abnormalities were found in the treated rats, consistent with the previous studies on probiotic strains (Endres et al., 2009). Specifically, treatment with *B. coagulans* IDCC 1201 did not cause weight loss or loss in feed intake (Table 5) or changes in appearance, behavior, or survival rate. At necropsy, no lesions were detected in any of the organs of the treated rats. Further histopathological examination was not performed based on the OECD/OCDE guideline No. 423. Based on these results, *B. coagulans* IDCC 1201 can be classified as Category 5 or Unclassified in the Globally Harmonized Classification System for Chemical Substances and Mixtures (GHS), an internationally recognized and harmonized chemical and mixture classification system, suggesting that *B. coagulans* IDCC 1201 causes relatively low or no acute toxicity hazard.

**Table 5.** The Change in Body Weight of the Rats Treated with *Bacillus coagulans* IDCC 1201

		Number of days after administration				
Group	Dose (g/kg BW^a^)	0	1	3	7	14
9-week-old	300	212.0 ± 6.3	234.3 ± 6.2	243.4 ± 5.3	251.4 ± 7.1	262.1 ± 11.0
	2 000	203.7 ± 5.5	224.9 ± 15.9	234.6 ± 11.1	245.5 ± 11.9	247.9 ± 13.5
10-week-old	300	228.3 ± 10.9	249.1 ± 15.3	257.7 ± 16.3	271.7 ± 14.1	280.7 ± 11.8
	2 000	244.8 ± 13.6	264.5 ± 16.2	271.7 ± 11.3	286.7 ± 13.2	304.5 ± 22.2

Based on the Student's *t*-tests using STATISTICA 7.0, there was no significant difference among the rats.

^a^BW, body weight.

### Evaluation of the Probiotic Properties of *B. coagulans*

The metabolic patterns of *B. cogaulans* IDCC 1201, that is, its strain-specific carbohydrate utilization patterns, were investigated using API 50 CH (Supplementary Table S6) (McLeod et al., 2008). The strain was found to use 28 carbohydrates among the 49 carbohydrates tested, including glycerol, l-arabinose, ribose, D-xylose, galactose, D-glucose, D-fructose, D-mannose, rhamnose, mannitol, salicine, cellobiose, maltose, lactose, melibiose, sucrose, trehalose, D-raffinose, amidon, gentibiose, D-turanose, α-methyl-D-glucoside, *N*-acetylglucosamine, amygdalin, arbutin, esculine, D-arabitol, and gluconate. The carbohydrate fermentation results were consistent with previous results showing that 85% of the tested 31 strains of *B. coagulans* was able to utilize D-galactose, D-fructose, D-glucose, glycerol, maltose, D-mannose, D-melibiose, *N*-acetylglucosamine, and D-trehalose (De Clerck et al., 2004).

**Fig. 3:**
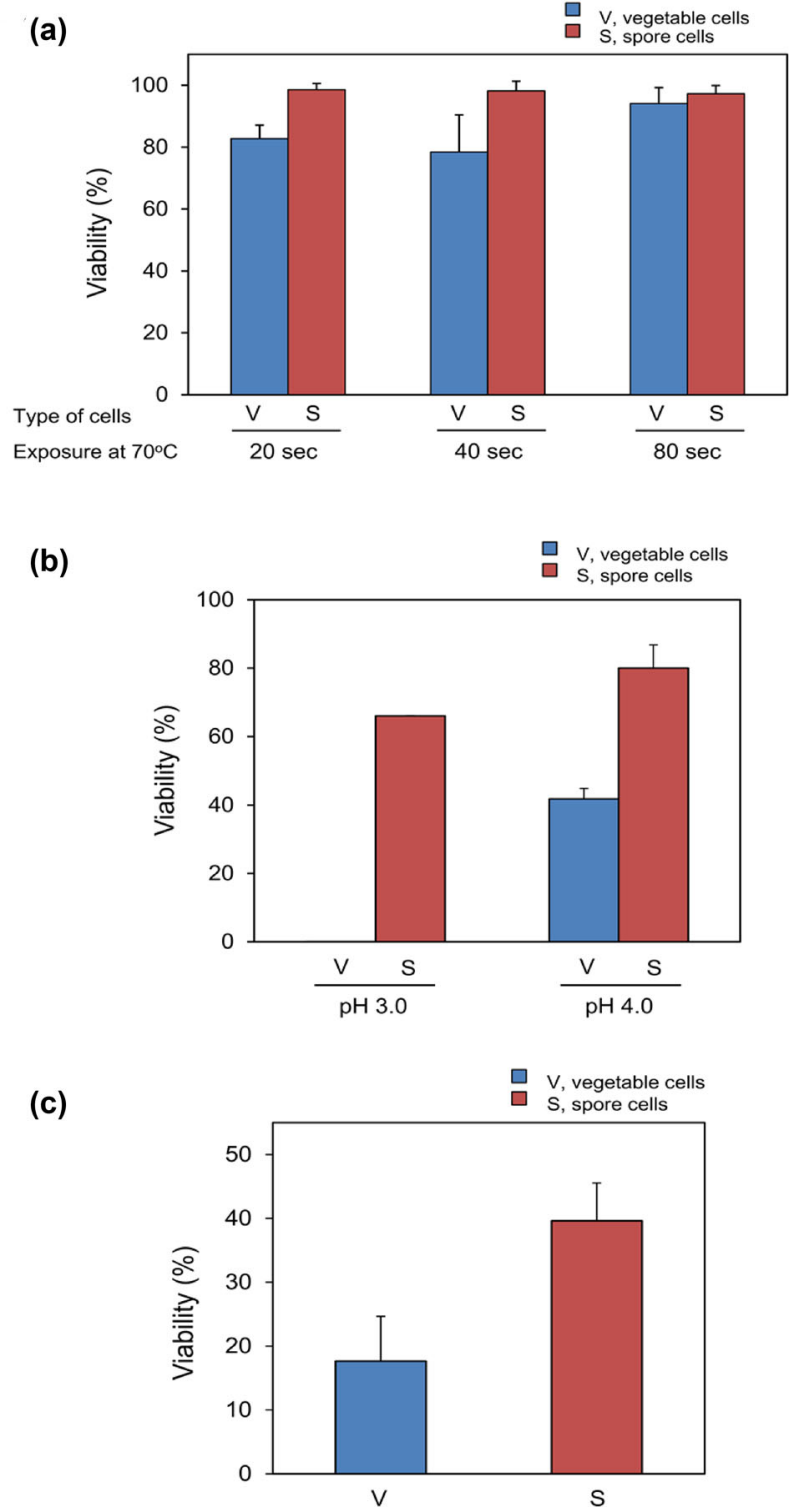
The Probiotic Properties of *B. coagulans* IDCC 1201 (a) heat, (b) acid and (c) bile tolerance.

The fundamental properties of probiotics, including heat, acid, and bile tolerance and hydrophobicity and autoaggregation involved in adhesion to intestines. Heat resistance is one of the primary parameters of processibility for probiotic bacteria to exert an appropriate function. Since the passage of surviving *B. coagulans* through the gastrointestinal tract is a vital function that must be exerted appropriately (Maathuis et al., 2010), we investigated the *in vitro* acid, heat, and bile tolerance of *B. coagulans* IDCC 1201 and its spore form (Fig. 3). After heat treatment at 70°C for 80 s, the viability of both *B. coagulans* IDCC 1201 and the spore were not significantly affected (P < 0.05). Notably, the initial cell concentration of 2.55 × 10^9^ CFU/ml was stably maintained at 2.40 × 10^9^ CFU/ml after heat exposure. Also, *B. coagulans* IDCC 1201 was observed to survive at pH 3 and 4 for up to 3 hr, achieving the eventual survival rate of 0.03% and 41%, respectively. However, it could not survive at a pH lower than 2. In general, probiotic bacteria is considered to have a tolerance for low pH when it can survive in pH 3 for 1.5–2 hr (Haldar & Gandhi, 2016). Meanwhile, the spores of *B. coagulans* IDCC 1201 showed significantly higher acid tolerance, showing about 66% of survival ratio at pH 3 for 3 hr. Therefore, spore form of *B. coagulans* exhibited high tolerance to pH in the present study.

After passing through the stomach, probiotic bacteria are released into the small intestine and come into contact with the bile, which possesses potent antimicrobial activity by disrupting cell membranes (Lorena et al., 2013). In this study, *B. coagulans* was slightly affected by 0.3% bile salts (Fig. 3). For example, approximately 15.8 billion *B. coagulans* cells and 4.3 billion spore cells were reduced to 4.7 million cells and after 6 hr of incubation of the strain, indicating approximately 0.03% cell viability. Unlike vegetative cells, spore cells were only a little affected, indicating 70.2% cell viability. It indicates a slight sensitivity of *B. coagulans* IDCC 1201 to bile compared to previous studies (Mirelahi et al., 2009; Ahire et al., 2011; Chackoshian & Shojaosadati, 2017).

Surface hydrophobicity and autoaggregation abilities are essential properties of probiotics because they enable probiotics to colonize the intestinal tract (De Souza et al., 2019; Krausova et al., 2019; Pan et al., 2017). *B. coagulans* IDCC 1201 exhibited approximately 17.5% of hydrophobicity and displayed 13.0%, 16.0%, and 29.1% of autoaggregation abilities after 1, 3, and 5 hr, respectively, which continually increased with time. The hydrophobicity and autoaggregation properties varied based on strains, culture media, and culture conditions (Lorena et al., 2013). For instance, the hydrophobicity of various probiotic bacteria, including *Lactobacillus* spp. and *Bacillus* spp., ranged from 6.1% to 87.4% (Martienssen et al., 2001; Shakirova et al., 2013; Krausova et al., 2019) or 0.3% to 68.8% (Shakirova et al., 2013). It was also reported that autoaggregation after 24 hr ranged from 21.7% to 69.7%, indicating considerable variation among the strains in the same taxonomy (Krausova et al., 2019). In summary, *B. coagulans* IDCC 1201 has demonstrated functional potential as a probiotic bacterial strain with respect to heat, acid, and bile tolerance and adhesion ability in the intestinal environment under *in vitro* conditions.

## Conclusion

The data in this study have demonstrated the safety of *B. coagulans* IDCC 1201 as a food additive. This strain was found to be safe based on genomic and phenotypic analyses. Furthermore, experiments did not uncover the strain's cell cytotoxicity or AOT. Finally, this strain has demonstrated its potential probiotic properties in the intestinal environment. Therefore, *B. coagulans* IDCC 1201 can be safely used for human consumption as a probiotic.

## Supplementary Material

kuab026_Supplemental_File
