# Supplementary material for: Genomic-, phenotypic-, and toxicity-based safety assessment and probiotic potency of Bacillus coagulans IDCC 1201 isolated from green malt
Source: J Ind Microbiol Biotechnol. 2021 Apr 27;48(5-6):kuab026. doi: 10.1093/jimb/kuab026 (PMC9113417; doi:10.1093/jimb/kuab026)

**Supporting Information**

**Genomic-, phenotypic-, and toxicity-based safety assessment and probiotic potency of *Bacillus coagulans* IDCC 1201 isolated from green malt**

Won Yeong Bang^1^, O-Hyun Ban^2^, Bo Som Lee^1^, Sangki Oh^2^, Chanmi Park^2^, Mi-Kyung Park^1^, Sung Keun Jung^1^, Jungwoo Yang^2*^, Young Hoon Jung^1,3*^

^1^ School of Food Science and Biotechnology, Kyungpook National University, Daegu 41566, Republic of Korea

^2^ Ildong BioScience, 17 Poseunggongdan-ro, Pyeongtaek-si, Gyeonggi-do, 17957, Republic of Korea

^3^ Institute of Fermentation Biotechnology, Kyungpook National University, Daegu 41566, Republic of Korea

* Corresponding author:

Jungwoo Yang

E-mail: yjw@ildong.com; Phone: 82-31-646-3114; Fax: 82-70-7500-2592

Young Hoon Jung

E-mail: [younghoonjung@knu.ac.kr](mailto:younghoonjung@knu.ac.kr); Phone: 82-53-950-5777; Fax: 82-53-950-6772

**Supplementary Table 1.** (A) Taxonomic information of *B. coagulans* IDCC 1201 acquired by 16S rDNA sequencing and (B) summary of the genome

| A | | | | |
| --- | --- | --- | --- | --- |
| Property | Term | | |  |
| Classification | Domain | | *Bacteria* |  |
|  | Phylum | | *Firmicutes* |  |
|  | Class | | *Bacilli* |  |
|  | Order | | *Bacillales* |  |
|  | Family | | *Bacillaceae* |  |
|  | Genus | | *Bacillus* |  |
|  | Species | | *Bacillus coagulans* |  |
| B | | | | |
| Property | | Statistics | | |
| Total genome size | | 3,649,442 bp | | |
| GC ratio | | 46.27% | | |
| No. of contigs | | 1 | | |
| No. of CDSs | | 3,473 | | |
| No. of rRNA genes | | 30 | | |
| No. of tRNA genes | | 84 | | |

**Supplementary Table 2.** Various species of *Bacillus coagulans* retrieved from the NCBI genome database for phylogenetic analysis in this study

| **Species** | **Strain** | **Genome assembly accession no.** | **Sources** | **Notes** |
| --- | --- | --- | --- | --- |
| *Bacillus coagulans* | 36D1 | GCA_000169195.2 | Mud | - |
| *Bacillus coagulans* | 2-6 | GCA_000217835.1 | Soil | - |
| *Bacillus coagulans* | XZL4 | GCA_000223155.1 | Soil | - |
| *Bacillus coagulans* | XZL9 | GCA_000333915.1 | Soil | - |
| *Bacillus coagulans* | H-1 | GCA_000333935.1 | Soil | - |
| *Bacillus coagulans* | CSIL1 | GCA_000482605.1 | - | - |
| *Bacillus coagulans* | GBI-30, 6086 | GCA_000756285.1 | Marketed probiotic product | - |
| *Bacillus coagulans* | P38 | GCA_000773655.1 | Soil | - |
| *Bacillus coagulans* | ATCC 7050 | GCA_000832905.1 | Dairy products | Type strain |
| *Bacillus coagulans* | HM-08 | GCA_000876545.1 | Healthy chicken intestine | - |
| *Bacillus coagulans* | NL01 | GCA_000988585.1 | Soil | - |
| *Bacillus coagulans* | S-lac | GCA_001039495.1 | Commercial probiotics | - |
| *Bacillus coagulans* | GED7749B | GCA_001546215.1 | Vagina | - |
| *Bacillus coagulans* | Unique IS-2 | GCA_001578455.1 | Human feces | - |
| *Bacillus coagulans* | B4100 | GCA_001587205.1 | Low pH sauce | - |
| *Bacillus coagulans* | B4099 | GCA_001587215.1 | Indian curry | - |
| *Bacillus coagulans* | B4098 | GCA_001587225.1 | Chinese tomato | - |
| *Bacillus coagulans* | B4096 | GCA_001587275.1 | Tomato supreme | - |
| *Bacillus coagulans* | BC-HY1 | GCA_001870065.1 | Digestive tract of healthy swine | - |
| *Bacillus coagulans* | HS243 | GCA_002266595.1 | Commercial food preparation | - |
| *Bacillus coagulans* | LBSC | GCA_002786555.1 | Soil | - |
| *Bacillus coagulans* | R11 | GCA_002946455.1 | Soil | - |
| *Bacillus coagulans* | LA204 | GCA_003184245.1 | Soil | - |
| *Bacillus coagulans* | ZB29 | GCA_003335145.1 | Milk | - |
| *Bacillus coagulans* | AF24-21 | GCA_003459145.1 | Human feces | - |
| *Bacillus coagulans* | AF24-19 | GCA_003459195.1 | Human feces | - |
| *Bacillus coagulans* | IDCC1201 | GCA_004114715.1 | Green malt | **-** |
| *Bacillus coagulans* | MA-13 | GCA_004359975.1 | Agricultural waste | - |
| *Bacillus coagulans* | DSM 2314 | GCA_006716385.1 | Soil | - |
| *Bacillus coagulans* | CICC 20138 | GCA_009936615.1 | Fermenter | - |
| *Bacillus coagulans* | LSBC-1 | GCA_010820925.1 | Soil | - |
| *Bacillus coagulans* | CGD018 | GCA_012851395.1 | - | - |
| *Bacillus coagulans* | CGI314 | GCA_012910695.1 | Laboratory |  |
| *Bacillus coagulans* | ASRS217 | GCA_013402775.1 | Rice straw | - |
| *Bacillus coagulans* | MGYG-HGUT-00191 | GCA_902364135.1 | Human gut |  |
| *Bacillus acidiproducens* | DSM 23148 | GCA_000374345.1 | Vineyard soil | Type strain, Outgroup for tree |

**Supplementary Table 3. Stability of the genome of *B. coagulans* IDCC 1201: Analysis of mobile elements (transposons, conjugal transfer proteins, and prophage regions)**

| No. | Prophage region length | Completeness (score) | Contig | Position | GC % | ATT_Site _showup | TRNA # | Total _protein # | Phage_hit protein # |
| --- | --- | --- | --- | --- | --- | --- | --- | --- | --- |
| 1 | 40.2Kb | incomplete(50) | 1201_1 | 1073219-1113470 | 42.98% | yes | 0 | 39 | 24 |
| 2 | 9.6Kb | incomplete(30) | 1201_1 | 1209868-1219532 | 44.86% | no | 0 | 10 | 6 |
| 3 | 46.4Kb | intact(150) | 1201_1 | 2703843-2750303 | 42.64% | yes | 0 | 54 | 35 |
| The prophage region 3 of the chromosome is similar to the thermophilic bacteriophage GVE2 (NC_ 009552)  There is no virulence gene or antibiotic resistance gene in these prophage regions.  The genome harbors 95 genes encoding transposases. No transposase was found in the 10-kb regions surrounding the virulence genes  Strain has no plasmid. The genome also does not encode conjugal transfer proteins. | | | | | | | | | |

**Supplementary Table 4. Analysis of genomic islands**

| PutativeGenomic Island | Gene Composition | Genomic Position | | GC deviation | Codon usage deviation | Putative  PAthogenicity Island | Putative Resistance Islands | Note |
| --- | --- | --- | --- | --- | --- | --- | --- | --- |
|  |  | Start | End |  |  |  |  |  |
| GI_1 | 1201_1_00020-1201_1_00022 | 22,371 | 27,749 | 0% | 37% | - | - | - |
| GI_2 | 1201_1_00214-1201_1_00231 | 235,799 | 253,485 | 44% | 33% | - | - | - |
| GI_3 | 1201_1_00256-1201_1_00262 | 283,728 | 291,718 | 28% | 14% | - | - | - |
| GI_4 | 1201_1_00393-1201_1_00400 | 431,591 | 441,258 | 27% | 9% | - | - | - |
| GI_5 | 1201_1_00414-1201_1_00433 | 453,563 | 472,091 | 70% | 55% | - | - | - |
| GI_6 | 1201_1_00773-1201_1_00796 | 826,505 | 848,081 | 41% | 68% | - | - | - |
| GI_7 | 1201_1_00924-1201_1_00928 | 975,285 | 981,687 | 50% | 66% | Strong | - | - |
| GI_8 | 1201_1_01028-1201_1_01048 | 1,066,614 | 1,080,560 | 36% | 72% | Strong | - | immunity repressor protein |
| GI_9 | 1201_1_01059-1201_1_01098 | 1,085,662 | 1,116,353 | 12% | 40% | - | - | - |
| GI_10 | 1201_1_01112-1201_1_01118 | 1,129,937 | 1,140,213 | 85% | 100% | - | - | - |
| GI_11 | 1201_1_01155-1201_1_01161 | 1,172,713 | 1,184,120 | 14% | 28% | - | - | - |
| GI_12 | 1201_1_01341-1201_1_01363 | 1,391,755 | 1,419,401 | 44% | 44% | - | - | - |
| GI_13 | 1201_1_01943-1201_1_01948 | 2,049,008 | 2,060,435 | 40% | 60% | - | Strong | Putative phage capsid protein, DNA_methyltransferase |
| GI_14 | 1201_1_02600-1201_1_02648 | 2,708,318 | 2,744,344 | 24% | 33% | - | - | - |
| GI_15 | 1201_1_03299-1201_1_03310 | 3,370,079 | 3,380,066 | 9% | 45% | - | - | - |
| GI_16 | 1201_1_03541-1201_1_03544 | 3,612,107 | 3,618,397 | 11% | 22% | - | Weak | Putative siderophore exporter |

16 genomic islands were predicted in this genome by the GIPSy program using the genome sequence of *B. coagulans* DSM 2314 as the reference genome.

**Supplementary Table 5. Enzymatic activities of *B. coagulans* IDCC 1201**

| Enzyme | *B. coagulans* |
| --- | --- |
| Alkaline phosphate | ++ |
| Esterase | + |
| Esterase lipase | - |
| Lipase | - |
| Leucine arylamidase | + |
| Valine arylamidase | - |
| Cystine arylamidase | - |
| Trypsin | - |
| α-chymotrypsin | - |
| Acid phosphatase | +++ |
| Naphthol-AS-BI-phosphohydrolase | ++ |
| α-galactosidase | +++ |
| β-galactosidase | +++ |
| β-glucuronidase | - |
| α-glucosidase | + |
| β-glucosidase | - |
| N-acetyl-β-glucosaminidase | - |
| α-mannosidase | - |
| α-fucosidase | - |

**Supplementary Table 6.** Fermentative profile of *B. coagulans* IDCC 1201 on API 50 CHL

| No. | Substrate | Result | No. | Substrate | Result |
| --- | --- | --- | --- | --- | --- |
| 1 | Glycerol | + | 26 | Salicin | + |
| 2 | Erythritol | - | 27 | Cellobiose | + |
| 3 | D-Arabinose | - | 28 | Maltose | + |
| 4 | L-Arabinose | + | 29 | Lactose | + |
| 5 | Ribose | + | 30 | Melibiose | + |
| 6 | D-xylose | + | 31 | Sucrose | + |
| 7 | L-Xylose | - | 32 | Trehalose | + |
| 8 | Adonitol | - | 33 | Inulin | - |
| 9 | β-Methyl-xylose | - | 34 | Melezitose | - |
| 10 | Galactose | + | 35 | d-raffinose | + |
| 11 | d-glucose | + | 36 | Amidon | + |
| 12 | d-fructose | + | 37 | Glycogene | - |
| 13 | d-mannose | + | 38 | Xylitol | - |
| 14 | l-sorbose | - | 39 | Gentiobiose | + |
| 15 | Rhamnose | + | 40 | d-turanose | + |
| 16 | Dulcitol | - | 41 | d-lyxose | - |
| 17 | Inositol | - | 42 | d-tagatose | - |
| 18 | Mannitol | + | 43 | d-fucose | - |
| 19 | Sorbitol | - | 44 | l-fucose | - |
| 20 | α-Methyl-d-mannoside | - | 45 | d-arabitol | + |
| 21 | α-Methyl-d-glucoside | + | 46 | l-arabitol | - |
| 22 | N-Acetyl-Glucosamine | + | 47 | Gluconate | + |
| 23 | Amygdaline | + | 48 | 2-keto-gluconate | - |
| 24 | Arbutin | + | 49 | 5-keto-gluconate | - |
| 25 | Esculine | + |  |  |  |
| +: positive reaction, -: negative reaction | | | | | |

**Supplementary Figure 1.** Genomic map of *B. coagulans* IDCC1201. Marked characteristics are shown from outside to the center; CDS on forward strand, CDS on reverse strand, tRNA (light green color), rRNA (red color), GC content and GC skew. Region that has higher value of GC percentage than average is described in exterior light green peak. Height of the peak describes the difference from the average GC percentage. The exterior light green peak describes the region that has higher G content while interior lavender peak describes the region that higher C content


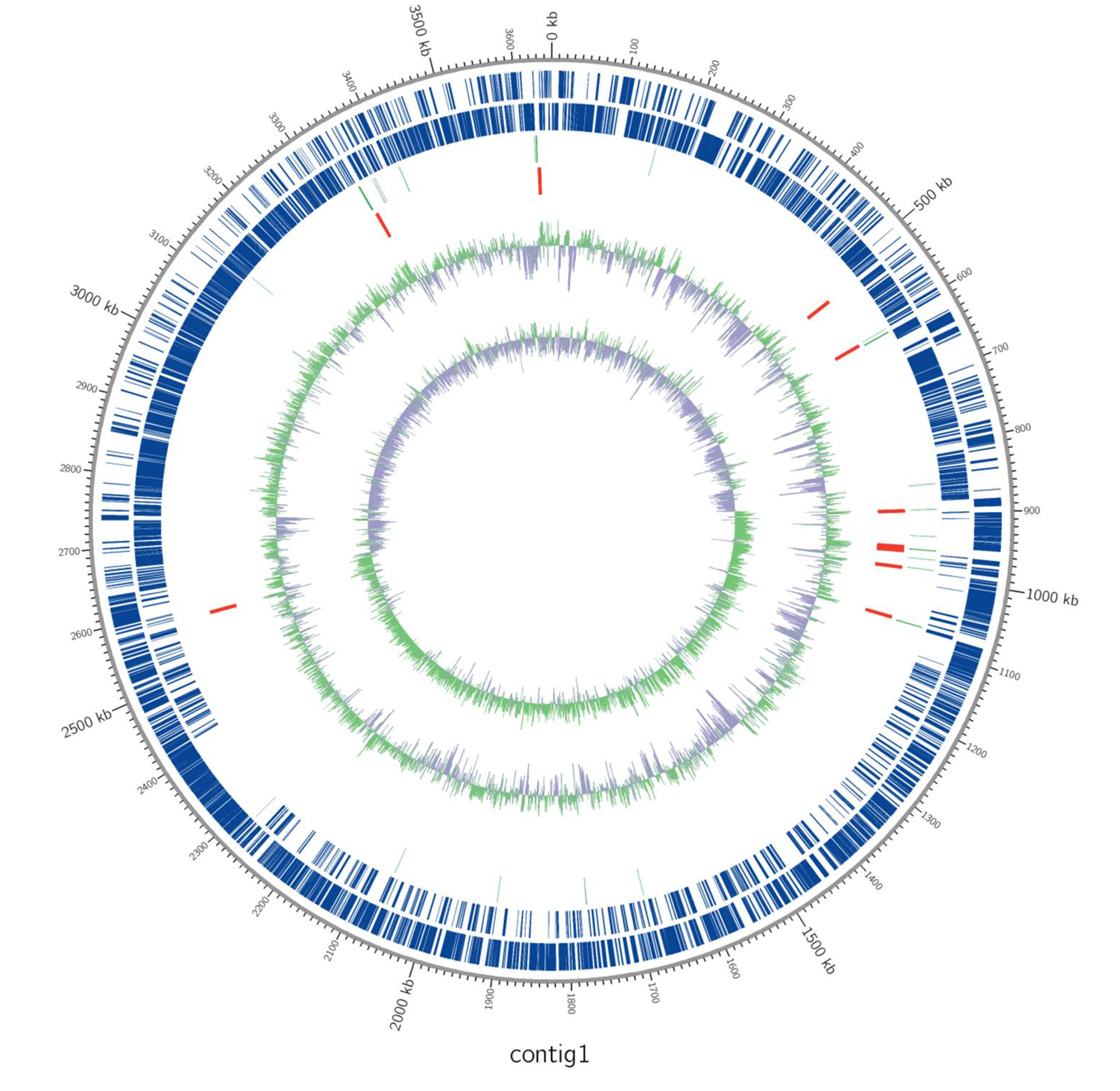


**Supplementary Figure 2**. Phylogenomic tree of the species *B. coagulans*. The tree was constructed using the amino acid alignments of 1,066 core genes with maximum likelihood approach. Numbers above branches show maximum-likelihood bootstrap supports from 500 non-parametric replicates (shown only if they were > 0.5). The tree was rooted by the *B. acidiproducens* DSM 23148 as an outgroup. The scale bar represents the number of substitutions per site.


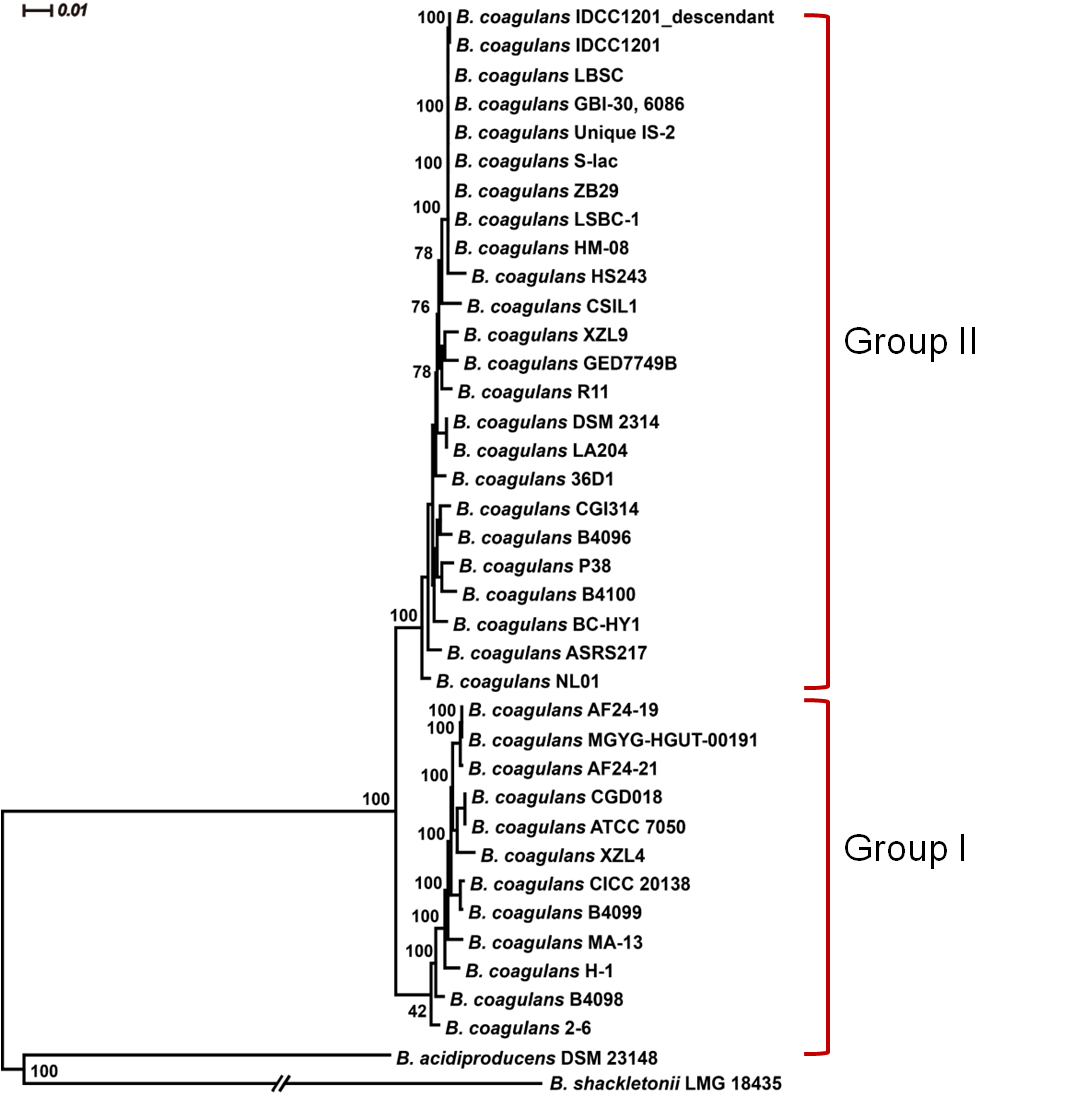

Supplement: kuab026_Supplemental_File [file kuab026_Supplemental_File.doc]
